# Biomass-derived carbon dots as fluorescent quantum probes to visualize and modulate inflammation

**DOI:** 10.1038/s41598-024-62901-7

**Published:** 2024-06-03

**Authors:** Mahima Kumar, Shanmugavel Chinnathambi, Noremylia Bakhori, Norhidayah Abu, Fatemeh Etezadi, Vaijayanthi Thangavel, Daniel Packwood, Easan Sivaniah, Ganesh N. Pandian

**Affiliations:** 1 Institute for Integrated Cell-Material Sciences (WPI-iCeMS), Institute for Advanced Study, Kyoto University, Kyoto, 616-8510 Japan; 2 Advanced Materials Research Centre (AMREC), SIRIM Berhad, Lot 34, Jalan Hi-Tech 2/3, Hi-Tech Park, 09000 Kulim, Malaysia; 3 Department of Medical Microbiology & Parasitology, School of Medical Sciences, Universiti Sains Malaysia, Health Campus, 16150 Kubang Kerian, Kelantan Malaysia; 4 Department of Molecular Engineering, Graduate School of Engineering, Kyoto University, Nishikyo-ku, Kyoto, 615-8510 Japan

**Keywords:** Carbon quantum dots, Biocompatibility, Biomarker, Inflammation, Imaging techniques and agents, Acute inflammation

## Abstract

Quantum dots, which won the Nobel Prize in Chemistry, have recently gained significant attention in precision medicine due to their unique properties, such as size-tunable emission, high photostability, efficient light absorption, and vibrant luminescence. Consequently, there is a growing demand to identify new types of quantum dots from various sources and explore their potential applications as stimuli-responsive biosensors, biomolecular imaging probes, and targeted drug delivery agents. Biomass-waste-derived carbon quantum dots (CQDs) are an attractive alternative to conventional QDs, which often require expensive and toxic precursors, as they offer several merits in eco-friendly synthesis, preparation from renewable sources, and cost-effective production. In this study, we evaluated three CQDs derived from biomass waste for their potential application as non-toxic bioimaging agents in various cell lines, including human dermal fibroblasts, HeLa, cardiomyocytes, induced pluripotent stem cells, and an *in-vivo* medaka fish *(Oryzias latipes)* model. Confocal microscopic studies revealed that CQDs could assist in visualizing inflammatory processes in the cells, as they were taken up more by cells treated with tumor necrosis factor-α than untreated cells. In addition, our quantitative real-time PCR gene expression analysis has revealed that citric acid-based CQDs can potentially reduce inflammatory markers such as Interleukin-6. Our studies suggest that CQDs have potential as theragnostic agents, which can simultaneously identify and modulate inflammatory markers and may lead to targeted therapy for immune system-associated diseases.

## Introduction

Carbonic nanomaterials such as nanodiamonds, fullerenes, nanotubes, graphene sheets, and CQDs have unique properties, inspiring extensive research for diverse technical applications. CQDs fluorescence emissions and optical properties have been widely studied and continue to generate interest in their potential applications^1^. Over the years, much research has been devoted to exploring the potential of semiconductor QDs, primarily due to their remarkable fluorescence emission properties that can be easily controlled and adjusted^2,3^. As a result, they have become an exceptionally valuable tool in biosensing and bioimaging, paving the way for new and innovative applications. Semiconductor QDs hold significant potential in biomedicine, bioimaging, and drug delivery, particularly with silicon QDs^4–7^. However, their production process involves heavy metals, which raises concerns about their environmental impact and potential health risks^8^. Therefore, exploring alternative production methods that could mitigate these issues and allow for the safe and effective use of QDs in clinical studies, bioimaging, drug delivery, and other such applications becomes essential. The advantages of CQDs, such as low cytotoxicity, favorable biocompatibility, high solubility, low cost, and chemical inertness, coupled with their similar fluorescence properties to semiconductor QDs, have made them a promising alternative^9^. As a result, CQDs exhibit the required potential to replace the semiconductor QDs.

Numerous studies have demonstrated the effective generation of CQDs using diverse biomass precursor sources^10–18^. Structurally, CQDs are composed of carbon, hydrogen, oxygen, and nitrogen and can be modified with polar functional groups such as carboxyl, amine, and hydroxyl. This improves their solubility and surface passivation, enhancing stability and performance. It also reduces surface defects and improves fluorescence. Functionalization with polar groups can also enhance their biocompatibility for biological applications. Doping heteroatoms or grafting other substances onto CQDs significantly enhances their quantum yield and strongly improves their attraction toward certain biomolecules. Amines like ethylenediamine, poly(ethylenediamine), and trimethylamine can enhance biological structure affinity, imaging resolution, and photobleaching resistance by attaching NH_2_ groups. Malaysia has taken a proactive approach to utilizing its natural resources sustainably and innovatively. By exploring the potential of biomass waste to produce nanomaterials, the country is promoting a “waste to wealth” approach in the field of nanotechnology. This helps reduce waste and contributes to the economy’s growth by developing new, eco-friendly technologies. Using waste from palm shells or seafood to create carbon-based QDs is an innovative and efficient way to produce sustainable energy.

CQDs can be synthesized through various methods, such as laser ablation, hydrothermal treatment, and microwave-assisted techniques^19^. Hydrothermal treatment is commonly used because it is easy, inexpensive, non-toxic, and sustainable. The hydrothermal method involves heating solutions in an enclosed chamber, inducing self-passivation. The optical and electronic properties of CQDs can be altered by precisely adjusting precursor concentration, dopant, pH, reaction time, temperature, and solvent type.

We chose to evaluate the capacity of CQDs for their capacity to stain inflammatory markers as recently, the world has witnessed the impact of cytokine storms during the COVID-19 pandemic and the emergence of new immune-related diseases. There is an increasing surge of evidence suggesting that inflammation, triggered by various stress stimuli, is a fundamental driver of several of these emerging diseases**.** Some of the major key inflammatory cytokine markers are- CCL-2, IL-6, and Tumor necrosis factor-alpha (TNF-α). The chemokine ligand 2 (CCL-2) is produced by various cell types, including endothelial cells, monocytes, fibroblasts, VSMCs, renal tubular epithelial cells, and neurons. CCL2-mediated cell migration plays a crucial role in the pathological processes of humans. Also, it is known to have a significant role in producing the immune response and contributing to the pathogenesis of immense diseases such as rheumatoid arthritis. CCL-2^20^ primarily interacts with its receptor, C–C chemokine receptor type 2 (CCR-2)^21^, which is expressed on the surface of target cells, including monocytes, macrophages, and certain T cells. Interleukin-6 (IL-6)^22^ is a proinflammatory cytokine that regulates essential immune processes in the human body, for instance, chronic inflammatory conditions, fever, cancer, and other autoimmune disorders. IL-6 has also been implicated in promoting inflammatory and auto-immune responses^23^. The JAK-STAT^24^ signaling pathway is activated in IL-6-induced inflammation, which triggers the transcription of genes that promote inflammation. Also, TNF-α^25^ a key proinflammatory cytokine, plays a key role in regulating the inflammatory pathways, activating immune cells, and stimulating an acute phase response. TNF-α is produced^26^ by various immune cells, such as T cells and macrophages, in response to infection, injury, and other stimuli. TNF-α binds to its receptors^27^, TNFR1 and TNFR2, on the surface of target cells. This binding then stimulates a series of intracellular signaling pathways, thereby causing inflammation. Quantum dot probes targeting pro-inflammatory markers could lead to enhanced diagnostic imaging, non-invasive monitoring, targeted delivery, localized treatments, and a deeper understanding of inflammatory pathways. Therefore, we chose to evaluate our CQDs for their capacity to visualize the inflammation and alter the endogenous expression of these pro-inflammatory markers.

CQDs were synthesized using a simple hydrothermal method with citric acid and palm/oyster shell wastes. All the synthesized CQDs have been characterized systematically. We investigated the impact of ethylenediamine on CQDs, exploring the effects of temperature, reaction time, and solvent pH on their photoluminescence properties. The CQDs were synthesized and tested for cytotoxicity. They were evaluated as staining agents for the inflammatory markers in various cell lines, including human dermal fibroblasts, HeLa cells, human induced pluripotent stem cells, chondrocytes, and cardiomyocytes. Additionally, we evaluated their staining capacity with the medaka fish egg model. Our CQDs possess remarkable optical properties and biocompatibility, signifying their immense potential for revolutionizing bioimaging applications^3^.

## Materials and Methods

### Materials

We collected palm kernel shells and oysters from two sources in Malaysia—Perusahaan Minyak Sawit Bintang and Fish Farm Kuala Muda—to use as carbon precursors for biomass waste. We procured a range of chemicals and solutions for the experiment, which were dissolved in ultra-pure water. We obtained ethylenediamine from Acros Organics in the USA, absolute ethanol from Merck in Germany, and hydrochloric acid, citric acid monohydrate, and sodium hydroxide from Merck in Germany as well. We bought phosphate buffer from Sigma-Aldrich in Germany, while the Hoechst 33342 Solution and BioTracker 650 Red Nuclear Dye were obtained from Sigma-Aldrich in the United States. We also used the Mito Tracker Green obtained from Invitrogen, a biotechnology company based in Japan.

### CQDs synthesis

Before usage, the palm kernel shell needs to undergo carbonization for a duration of three hours at a temperature of 600 °C while being exposed to N_2_ at a flow rate of 50 cc per minute. The temperature should gradually increase by 10 °C per minute. The oyster shells were crushed by an industrial mixer and sifted to a size smaller than 500 µm.

The synthesis steps for carbonized palm kernel shell (CPKS) and oyster shell to produce CQDs are outlined below: A solution containing EDA (0.2 mL), 1.0 M NaOH (3 mL), and 15 mL of deionized water was prepared, and then 0.3 g of CPKS (Quantum yield:2.5%) or 0.3 g of oyster shell (Quantum yield:1.5%) were added. Using a chemical-based technique, 3.0 g of citric acid (CA) and 1 mL of EDA were mixed in 40 mL of deionized water to prepare the CA-based CQDs (Quantum yield: 22%). The mixtures were stirred vigorously for a period of 30 min. Mixtures were transferred to a Teflon-lined stainless-steel reactor and subjected to a hydrothermal reaction at 160 °C for 4 h. After completion, the CQDs were filtered using a 0.22 µm filter syringe disc. The filtration process was repeated twice to eliminate particles that were larger in size. The CQDs were purified overnight using a 3.5 kDa dialysis tube and then rotavaporated at 70 °C for one hour to remove excess water. The CQDs were then kept at -20 °C for freeze-drying purposes to obtain the solid form of CQDs. For further usage, the CQDs were diluted in deionized water as desired.

### Characterization

The JASCO FP-8300 Fluorescence Spectrometer was used to measure the CQDs’ emission spectra between 300 and 600 nm were observed. The emission spectra were recorded at a rate of 1000 nm per minute using various excitation wavelengths. Each sample was diluted in 1.0 mL of water overall. A Hamamatsu Photonics Quantaurus-QY C11347-11Absolute PL Quantum Yield Spectrometer was used to measure the quantum yields, and the integrated measurement software was used to calculate them. The PerkinElmer Lambda 35 spectrophotometer from the United States recorded absorbance between 200 to 700 nm. The functional groups on the CQDs were identified using a Fourier-transform infrared (FTIR) spectrophotometer (Thermo Scientific, USA). The particle size was unambiguously determined through the use of a high-resolution transmission electron microscope (HR-TEM) from Philips in the USA. The dynamic light scattering method measured the particle size in the water (Malvern Zetasizer, UK). The CQDs’ surface chemistry was analyzed by Omicron Nanotechnology in Germany using X-ray photoelectron spectroscopy (XPS).

### Culture and maintenance of human dermal fibroblasts and HeLa cells

The culture and maintenance of primary mammalian cell lines- HeLa, Human dermal fibroblast, and Human untreated/control chondrocyte cells were performed with an early passage following the standard protocol. The tissue culture supplies were bought from Fisher Scientific. The HeLa cells were cultured in a 60 mm tissue culture plate whereas HDFs and chondrocyte cells were cultured in a 75 cm^2^ flask and kept at controlled conditions at 37 °C temperature, maintaining humidity with 5% CO_2_. The cells were grown DMEM culture medium by Thermo Fisher Scientific Inc., supplemented with 5000 U/mL penicillin, 50 mg/mL streptomycin, and 10% FBS. Furthermore, the cells were detached with trypsin(1X) and were plated on a 96-well plate with a seeding density of 5000 cells per well for the cytotoxicity assays. Following the 24 h pre- incubation, the cells were treated with CQDs. The Cell Counting Kit-8 was used to detect the percentage viability of cells, kept at an incubation period of 24 and 48 h (Dojindo Laboratories, Osaka, Japan). The absorbance was measured at 450 nm, keeping 650 nm as the reference wavelength using a microplate reader (MTP-880Lab; Corona, Hitachinaka, Japan). The mean ± standard deviation was utilized to present the results of the cell viability experiments, which were conducted in three biological replicates.

### Culture and maintenance of human induced pluripotent stem cells

The 253G1 cell line of human-induced pluripotent stem cells (hiPSCs) were sourced from the RIKEN BioResource Center (Japan). The cultivation process followed the established protocol, as described earlier^31^. In brief, feeder-free hiPSCs were seeded onto laminin 511 E8 fragments-coated plate (iMatrix511 Silk, MATRIXOME, NP892-021) and were maintained using a TeSR-E8 culture medium (STEMCELL TECHNOLOGY, USA). Incubation conditions included a humidified atmosphere with 95% air and 5% CO_2_ at 37 °C. Cells were sub-cultured upon reaching 70% confluency, and the cell medium was refreshed daily. The hiPSCs were visually inspected using an IX71 inverted microscope (Olympus) equipped with a DP71 camera (Olympus).

### Differentiation and maintenance of cardiomyocytes

hiPSCs to cardiomyocytes (iCMs) differentiation protocol was optimized previously^28,29^, Briefly, hiPSCs to mesoderm induction was started upon reaching 90–100% confluency by adding 12 µM CHIR99021 (Wako, Japan) to the differentiation medium consisting of B-27 supplemented-RPMI (containing glucose, no insulin, Thermo Fisher Scientific, NY, USA). CHIR99021 was removed exactly after 24 h. Followed by one day rest, on day 3 of differentiation the WNT inhibitor (2 µM XAV939, Wako) was added to the differentiation medium for 48 h. On day 7 of differentiation, the differentiation medium was replaced with B-27 supplemented RPMI containing insulin (Thermo Fisher Scientific, NY, USA), and the medium was changed every other day. The cells were expected to start beating between the eighth and fifteenth day of differentiation.

### Imaging studies in cells

For the visualization of accumulation and cellular uptake of CQDs in cells, we used a CLSM (FluoView FV10i, ZEISS) excited with a 405 nm laser. We utilized a 488 nm laser to target the mitochondria, and a 640 nm/405 nm laser to precisely focus on the nucleus. Confocal imaging was observed by seeding HeLa, dermal fibroblast, hiPSc, and cardiomyocytes in a 35 mm glass bottom dish with 80% confluency. After 24 h, 100 ug/ml of CQDs were added to the dish to achieve the final concentration. Further, incubating for 24 h, and giving 2-3 times wash with PBS, all the cells were fixed in 4% paraformaldehyde for 15 min before imaging. To enhance the cellular uptake and the fluorescence efficiency live cell imaging was also performed. The images were selected out of the best. The cell nuclei stains were diluted in cell media and were labeled using either Hoechst 33342 or BioTracker 650 Red Nuclear Dye. For the live cell imaging, after giving the wash with PBS, the nucleus and other stains were diluted in a DMEM fluorescent medium, which reduces the background efficiency. After the cellular imaging, the fluorescence intensity of untreated and treated (Inflamed) dermal fibroblasts and chondrocytes was calculated with the help of Image J.

### Medaka fish cytotoxicity study

We conducted in vivo experiments using medaka fish (*Oryzias latipes)* embryos. We procured mature fish from Higo-pet in Kyoto Prefecture and housed them in a glass aquarium filled with fresh water at a temperature of 25 °C, following a 14 h light cycle and a 10 h dark cycle. We monitored and treated triplicate eggs on a 96-well plate when they were 4 days old and checked the larvae every day until day 28, transferring them to a separate tank on day 17.

### Artificial induction of inflammatory model in human chondrocyte cells

Normal Human Knee Articular Chondrocytes (NHAC-kn) cell line was purchased from Lonza (Catalog no: CC-2550). For this experiment, cells were seeded in a 6-well plate, where the inflammatory stress was induced by replacing the medium of chondrocyte reagent pack™ subculture agents from Lonza (Catalog no: CC-3233) with a cell medium containing recombinant TNF-α protein(10 ng/ml) incubated for 24 h. Further, the cells were treated with different carbon QDs (CPKS, CA, Oyster) for the next 24 h. After incubation, the cells were detached, trypsinized, and collected for RNA Isolation using (Qiagen RNeasy Plus Mini Kit), performed cDNA using the Reverse Transcription kit, and the expression levels of TNF-α, IL-6, and CCL2 inflammatory cytokines were then measured with Real Time-qPCR (TaqMan Gene Expression Assay).

## Results

**Figure 1:**
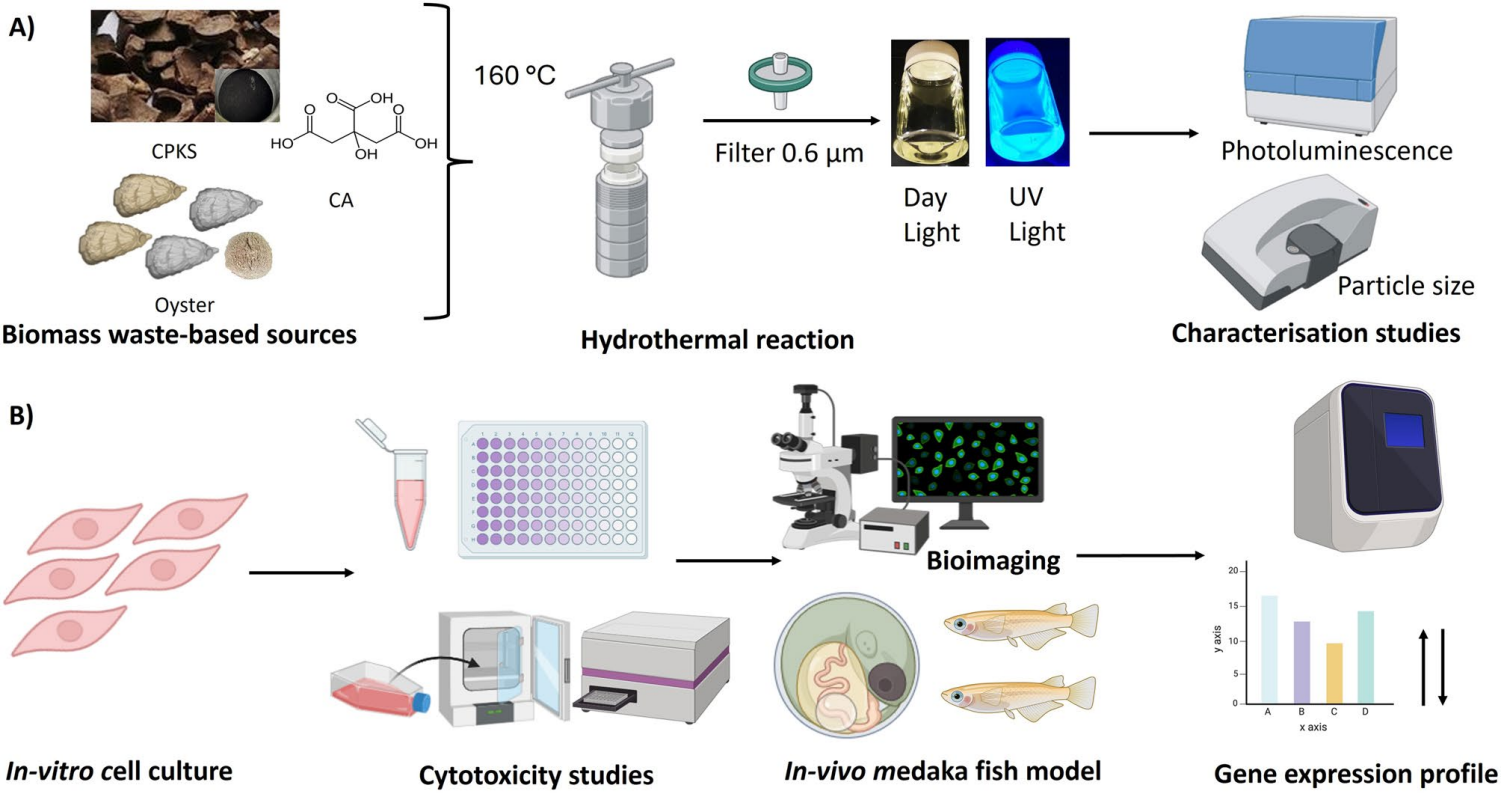
(**A**) Synthesis and characterization of Carbon QDs using CPKS, oyster shells, and citric acid sources. (**B**) Steps to treat QDs for Biological Applications. This figure was created with the assistance of Biorender.com.

CQDs were synthesized successfully from CA, CPKS, and oyster shells through a hydrothermal reaction. The CQDs exhibited fluorescence after being exposed to UV light, and their potential application as a biomarker for cellular aging has been explored. The CQDs synthesis process and application of these biomarker agents are illustrated in the diagram (Fig. 1).

### Characterization of the CQDs

**Figure 2:**
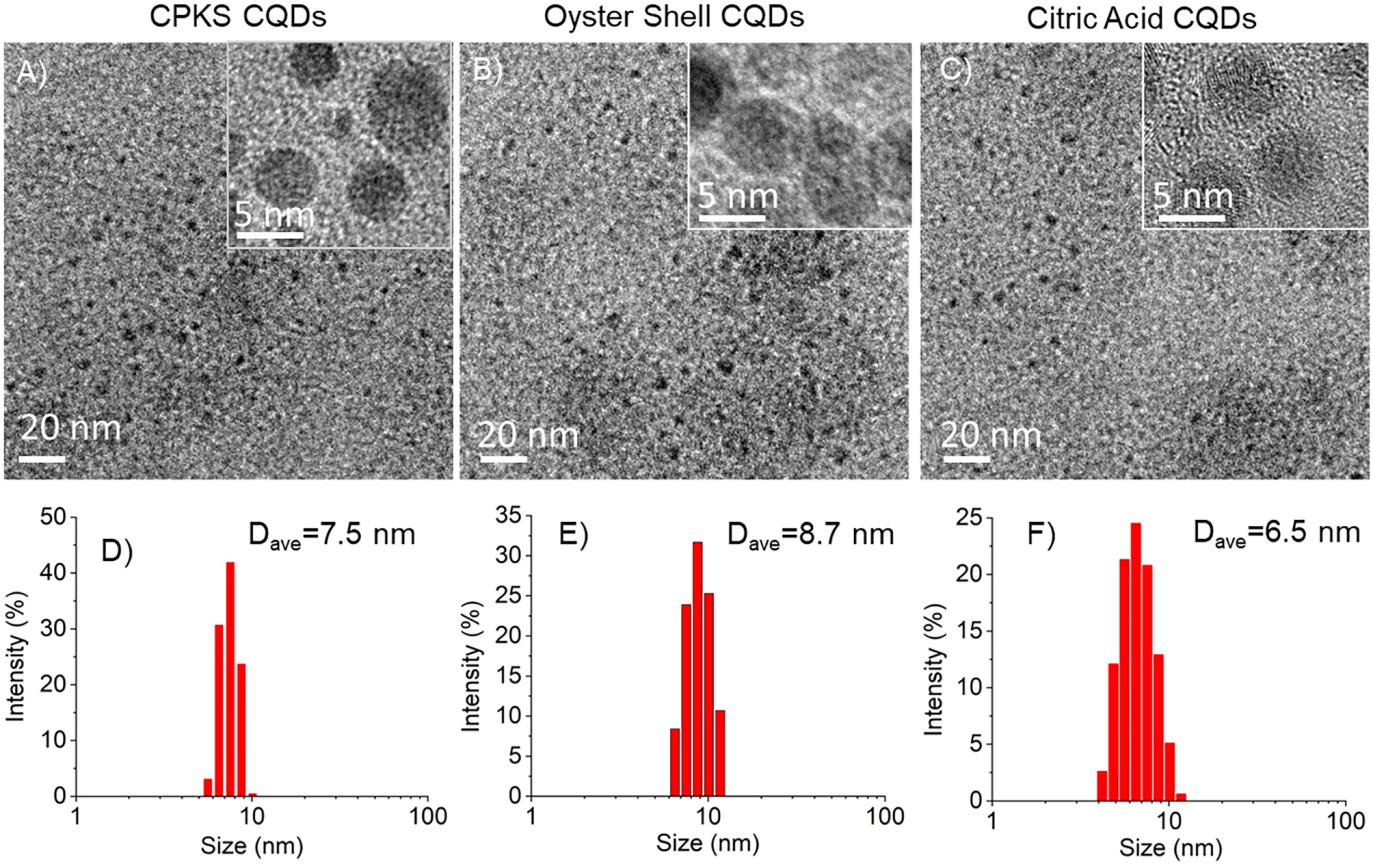
TEM images and dynamic light scattering sizes of carbon quantum dots synthesized from CPKS, oyster shells, and citric acid. The Inset images represent the high-resolution TEM images.

High-Resolution Transmission Electron Microscopy (HR-TEM) was employed to accurately determine the morphological structure of the CQDs. The CQDs were dropped onto the carbon grid and left to dry at room temperature (Fig. 2). The images presented in Fig. 2 A–C and insets demonstrate that the CQDs possess a well-defined spherical structure and are uniformly dispersed without any signs of clustering or aggregation. The size of the CDs in water was determined by dynamic light scattering, revealing a size of 7.5 nm for CPKS, 8.7 nm for Oyster, and 6.5 nm for CA (Figs. 2D–F). FT-IR (Figure S1) and XPS (Figure S2) analyses are included in supporting information.

**Figure 3:**
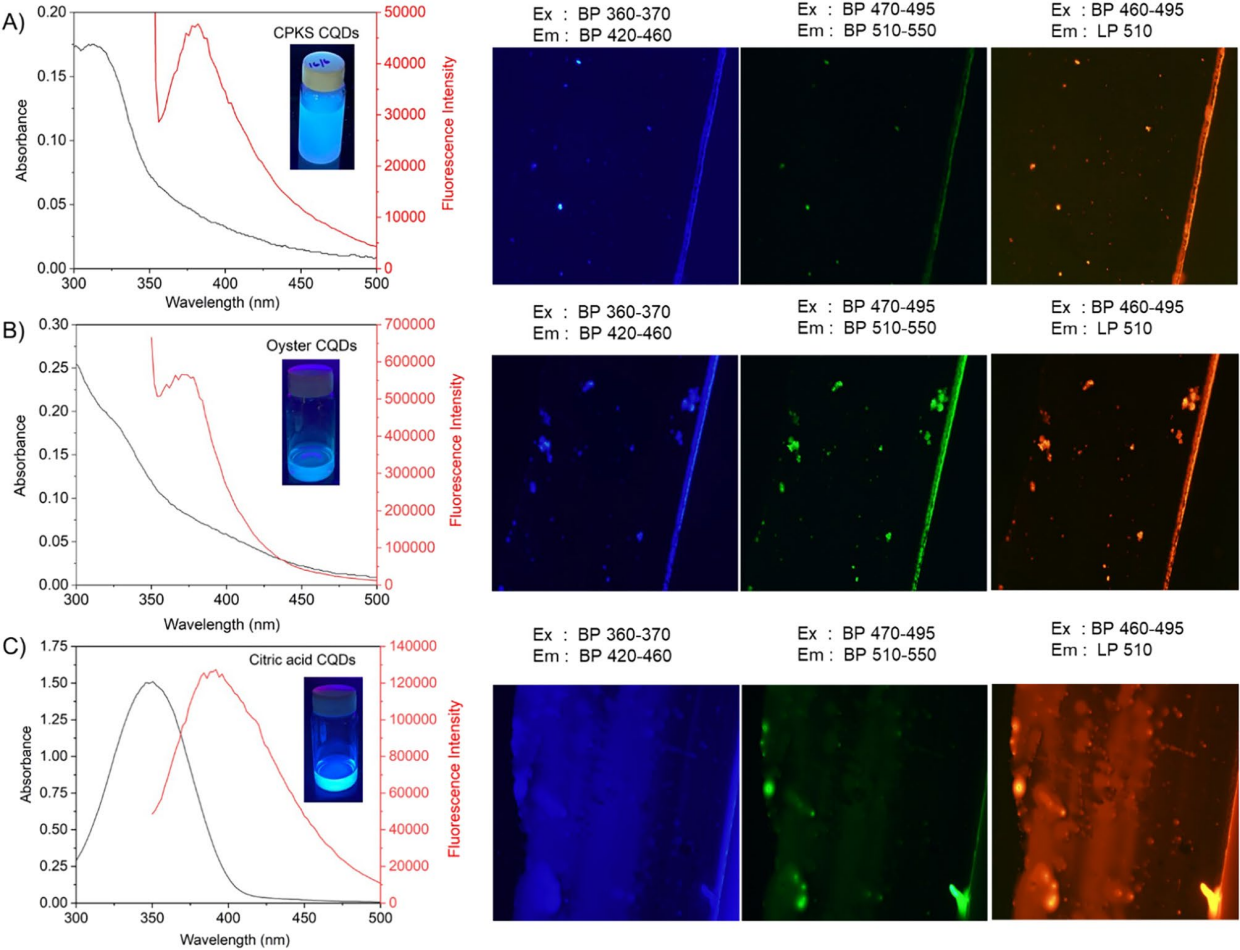
UV–visible absorption and fluorescence emission spectra of CPKS QDs (**A**), oyster QDs (**B**), and citric acid QDs (**C**) and their fluorescence images with three different excitations and emission band passes. The QDs are in liquid form on the glass and covered by a coverslip. The inset shows QDs fluorescence under 365 nm UV light excitation.

In order to comprehend the optical properties, we conducted an analysis of the CQDs through the use of absorption and fluorescence spectroscopy. The CQD’s fluorescence and absorption spectra are in Figures. 3A–C shows that they absorb light between 300 and 400 nm and emit it between 350 and 500 nm. The photograph of CQDs exposed to UV light is shown in the inset. All CQDs showed non-obvious UV–visible absorption spectra with a small shoulder at 280 nm, attributed to the presence of C = C bonds caused by the π-π* electron transition. Similarly, the peak for citric acid was observed at 345 nm. These peaks were ascribed to the n-π* transition of the non-bonding electrons in C–C, C-N, and C = O.

To capture the fluorescence images, the CPKS-based CQDs were carefully dropped onto a microscopic glass slide with a coverslip, as illustrated in the right-side panel of Fig. 3. During the observation of fluorescence emission, we utilized three distinct excitation filters: blue, green, and red. In terms of light intensity, blue channels outperform green and red channels. The results obtained are consistent with the fluorescence emission spectra of CQDs based on CPKS (Fig. 3A). We also observed CQDs synthesized from a combination of oyster shells and CA (Fig. 3B, C).

### Cytotoxicity and localization studies in various cell lines

To assess the biocompatibility of CQDs, we conducted cell viability and imaging studies using various cell lines, including fibroblast, HeLa, human induced pluripotent stem cells (hiPSc), and cardiomyocytes. As a result, after 24 h of incubation, HeLa cells remained almost 100% viable at the concentration of 1 mg/mL of CQDs from CPKS, oyster shell, and CA, as no cytotoxic effects were observed, as shown in Figure S3. Intriguingly, CPKS exhibits no toxicity even after 48 h of incubation, indicating its potential as a safe and effective theranostic agent. Regarding cadmium-based QDs, no toxicity was observed after 24 h. However, 15% toxicity was seen at increased concentration (1 mg/mL) after 48 h. Cell viability studies show functionalized CQDs are a more promising alternative to heavy-metal quantum dots, as they pose a lower risk.

CQDs synthesized using CPKS showed no significant cytotoxicity for the hiPSc culture even at a high 200 μg/mL concentration for 24 h. However, no toxicity was observed even after 48 h. After conducting experiments, it was found that oyster shell-based CQDs are safe to use up to 1 mg/mL for 24 and 48 h, as no cytotoxicity was observed (Figure S4). Within 24 h, citric acid CQDs showed no signs of toxicity, even up to 600 μg/mL. This suggests that citric acid CQDs can be used safely without being detrimental. However, when treated at 1000 μg/mL, 30% toxicity was shown. After 48 h, hiPSc cells showed more than 60% cytotoxicity from 400 to 1000 μg/mL. Figure S5 displays the process of cellular uptake of CQDs by hiPSc cells. The hiPSc cells exhibit excellent CQD uptake throughout their structure. Our experimental findings indicate that 200 μg/mL was the optimal concentration for CPKS and oyster CQDs. In contrast, CA-based CQDs showed exceptional fluorescence capacity and low toxicity at only 100 μg/mL concentrations. We observed no changes in cell morphology after 24 h incubation with CQDs. The cell viability studies demonstrate that CQDs at concentrations lower than 200 μg/mL are completely non-toxic to hiPSc cells.

CPKS-based CQDs did not affect heart cells (cardiomyocytes) when used up to 600 μg/mL for 48 h. However, when used at higher doses of 800 μg/mL and 1000 μg/mL for the same period, they cause 25% toxicity to these cells. We found that oyster-derived CQDs exhibited no toxicity at concentrations up to 800 μg/mL after 24 h (Figure S6). However, when we used a higher concentration of 1000 μg/mL, 10% of the cell population died. After 48 h, the CQDs showed 50% toxicity at 1000 μg/mL concentrations. CA-based CQDs showed no toxicity till 600 μg/mL. However, at a concentration of 1000 μg/mL, 35% of the cells showed toxicity. Figure S7 visually represents the efficient uptake of CQDs by cardiomyocyte cells. The image illustrates the widespread distribution of CQDs throughout the cells of the cardiomyocyte, demonstrating their effective cellular uptake. For CPKS and oyster CQDs, the quantity of 200 μg/mL was used, while for citric acid CQDs, only 100 μg/mL was used owing to their exceptionally high fluorescence capacity. After 24 h of incubation with CQDs, there were no observable changes in cell morphology, indicating that CQDs may have a low cytotoxicity profile. The study demonstrated that using CQDs at a concentration of 200 μg/mL did not cause any harm to the cardiomyocyte cells, indicating their non-toxicity.

**Figure 4:**
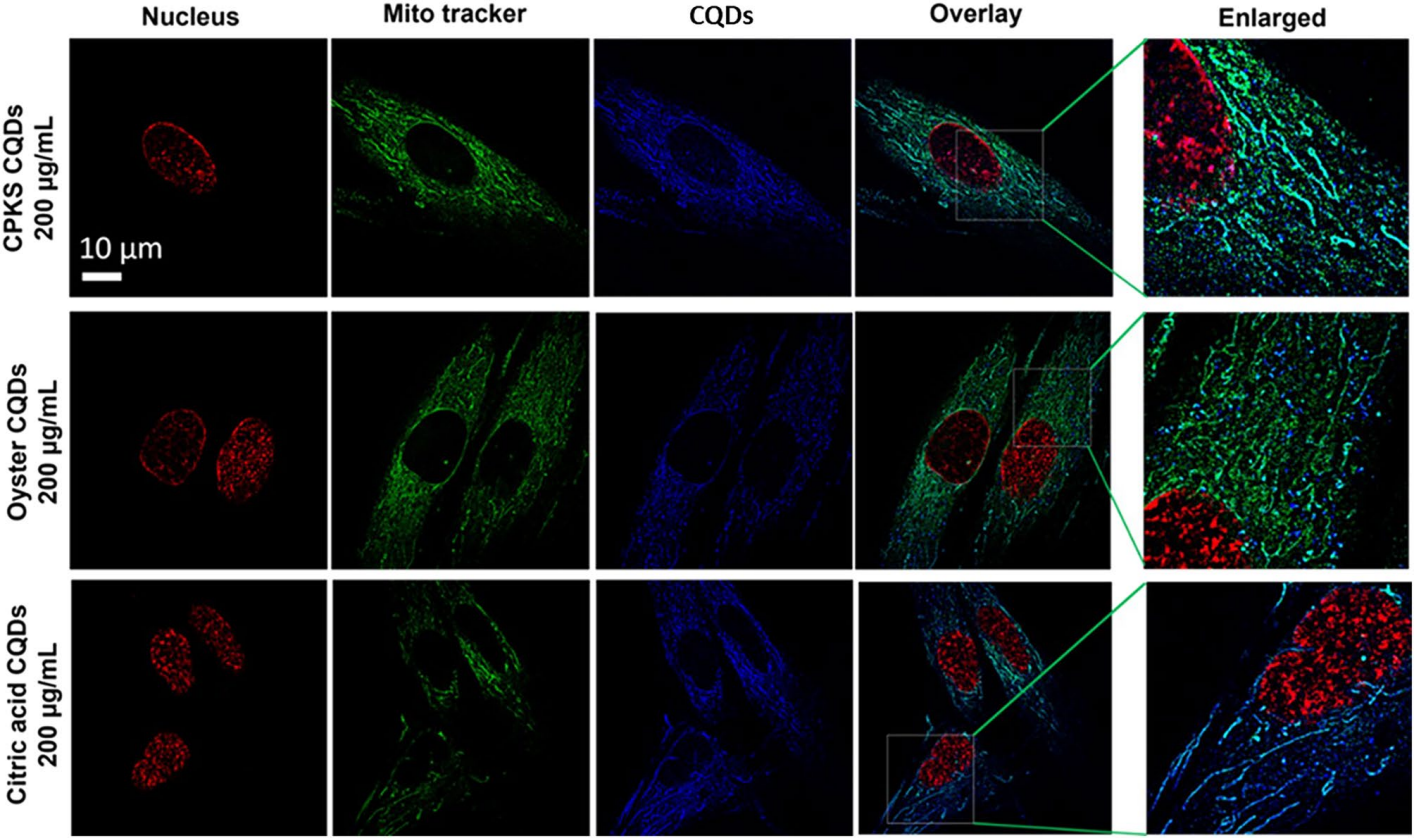
Fibroblast cells were treated with carbon quantum dots (CQDs) and imaged with CSLM. Staining with Bio Tracker dye (red) and Mito Tracker (green) shows the nucleus and mitochondria uptake, respectively, in relation to CQDs (Blue). The last two columns show an overlap and enlarged image.

**Figure 5:**
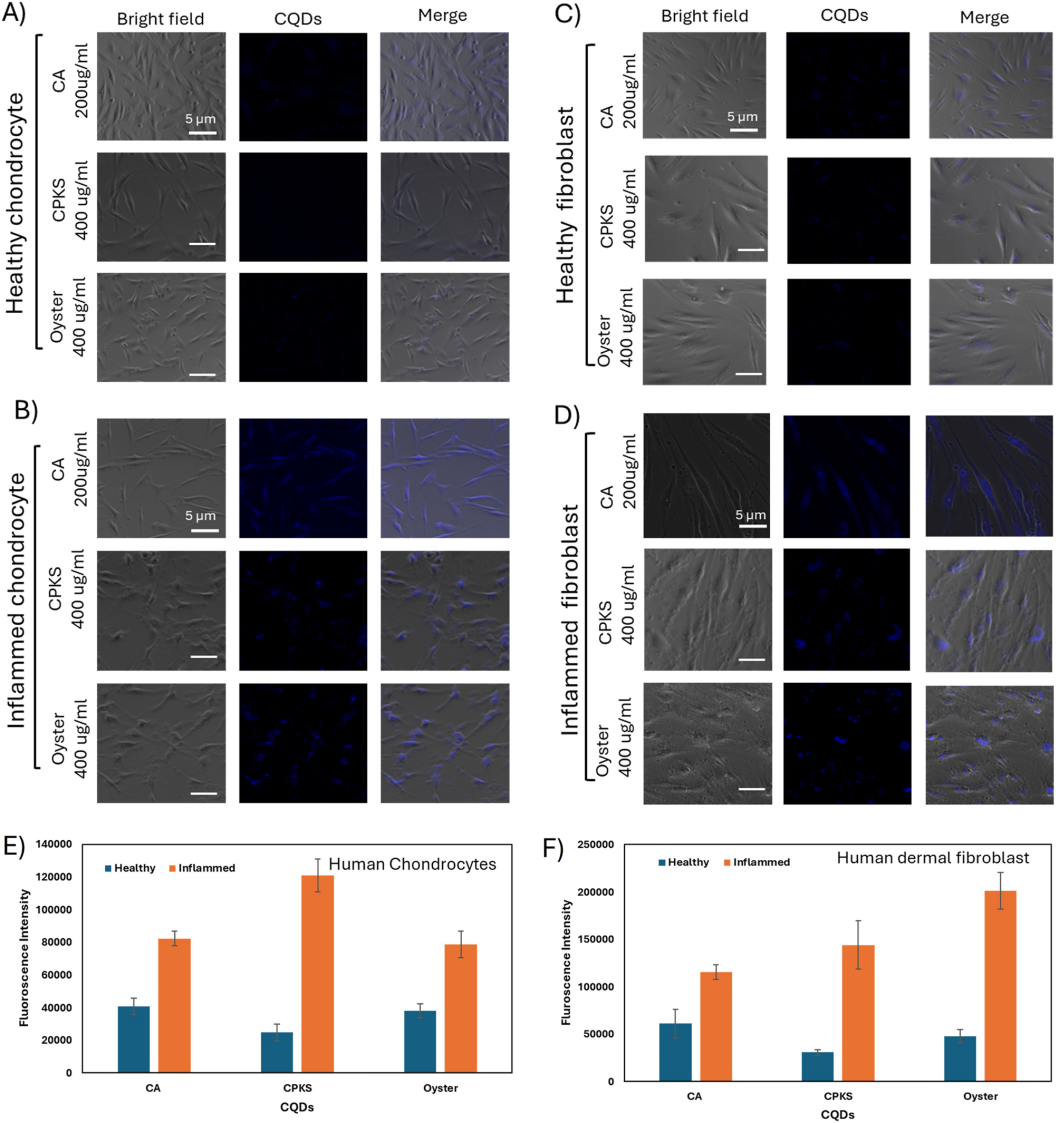
(**A**, **B**) Fluorescence images showing cellular uptake with CQDs for healthy and inflamed chondrocyte cells. (**C**, **D**) Fluorescence images showing cellular uptake with CQDs for healthy and inflamed dermal fibroblast cells. (**E**, **F**) Fluorescence intensity was measured using Image J for Human chondrocyte and Human dermal fibroblast cells, respectively. We selected an equal area per cell and measured fluorescence intensity (n = 3).

We examined the cellular uptake and accumulation of CQDs using human dermal fibroblasts and chondrocyte cells (Fig. 4). Our objective was to determine if CQDs, when used as non-toxic bioimaging agents, can facilitate differentiating the untreated and inflamed cells. At first, one group of cells was treated with TNF-α, whereas the other group was untreated (healthy) cells. Cells were further treated with Palm kernel shell, oyster, and CA CQDs with a concentration of 200 μg/mL, and as a result, we observed enhanced uptake of quantum dots for inflamed human dermal fibroblast and chondrocyte cells. In contrast, the untreated group of cells has significantly less uptake, almost negligible. Along with the cellular uptake and bioimaging, fluorescence intensity was calculated using Image J software (n = 3) (Fig. 5). As a result of confocal microscopic studies, when the cells were treated with tumor necrosis factor-α, showed a higher fluorescence uptake efficiency. This is because of the increased reactive oxygen species (ROS) level in inflamed cells^30^.

Moreover, most of the material was present around the cell nucleus. Also, to look for subcellular localization of quantum dots through confocal laser scanning microscopy, the Mito-tracker staining dye was utilized, and some QDs were also observed to overlap on the mitochondria. The cell nucleus is represented by red, while the mitochondria are represented by green. CQDs can easily enter the cells through endocytosis^31^ Studying the interaction mechanism between nanoparticles and cell uptake is essential in NP-based targeted drug delivery applications and may lead to designing more efficient nanomedicines (Fig. 4).

**Figure 6:**
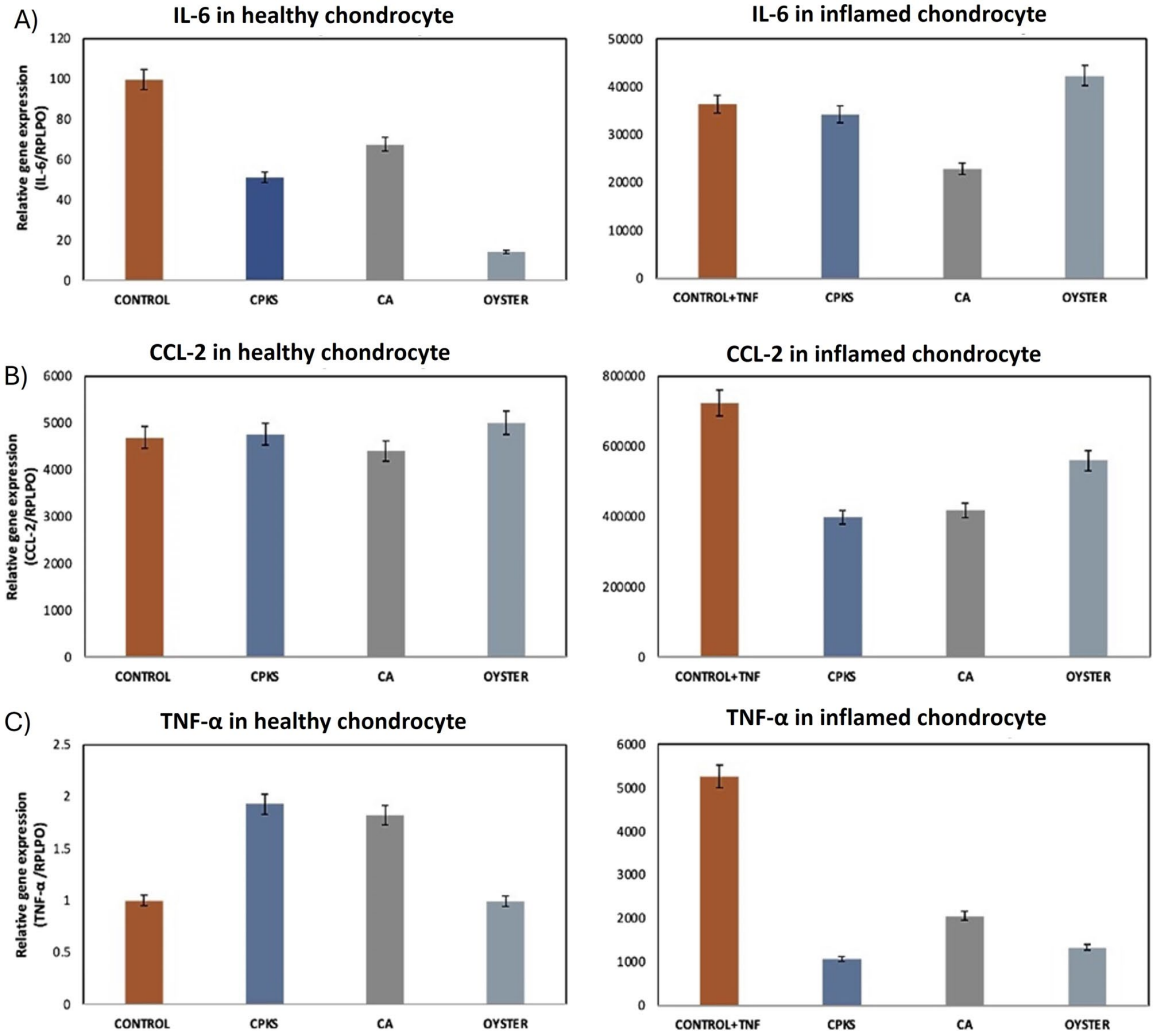
Human chondrocyte cells were treated with TNF-α (10 ng/ml), and the expression of inflammatory genes, typically IL-6 (**A**), CCL-2 (**B**), and TNF-α (**C**), was checked by qRT-PCR, taking RPLPO as a housekeeping gene. Standard error 5%.

In experiments involving untreated (healthy) and inflamed chondrocyte cells with the three CQDs, it was observed that out of the three CQDs, citric acid-derived quantum dots reduced the expression of inflammatory markers more efficiently, particularly the pro-inflammatory cytokine marker IL-6 in inflamed cells. This suggests that CA-derived CQDs could be harnessed to not only differentiate between control/untreated and inflamed cells but also alter their gene expression and may ensue anti-inflammatory properties (Fig. 6).

### In vivo cytotoxicity assay using medaka fish egg model

**Figure 7:**
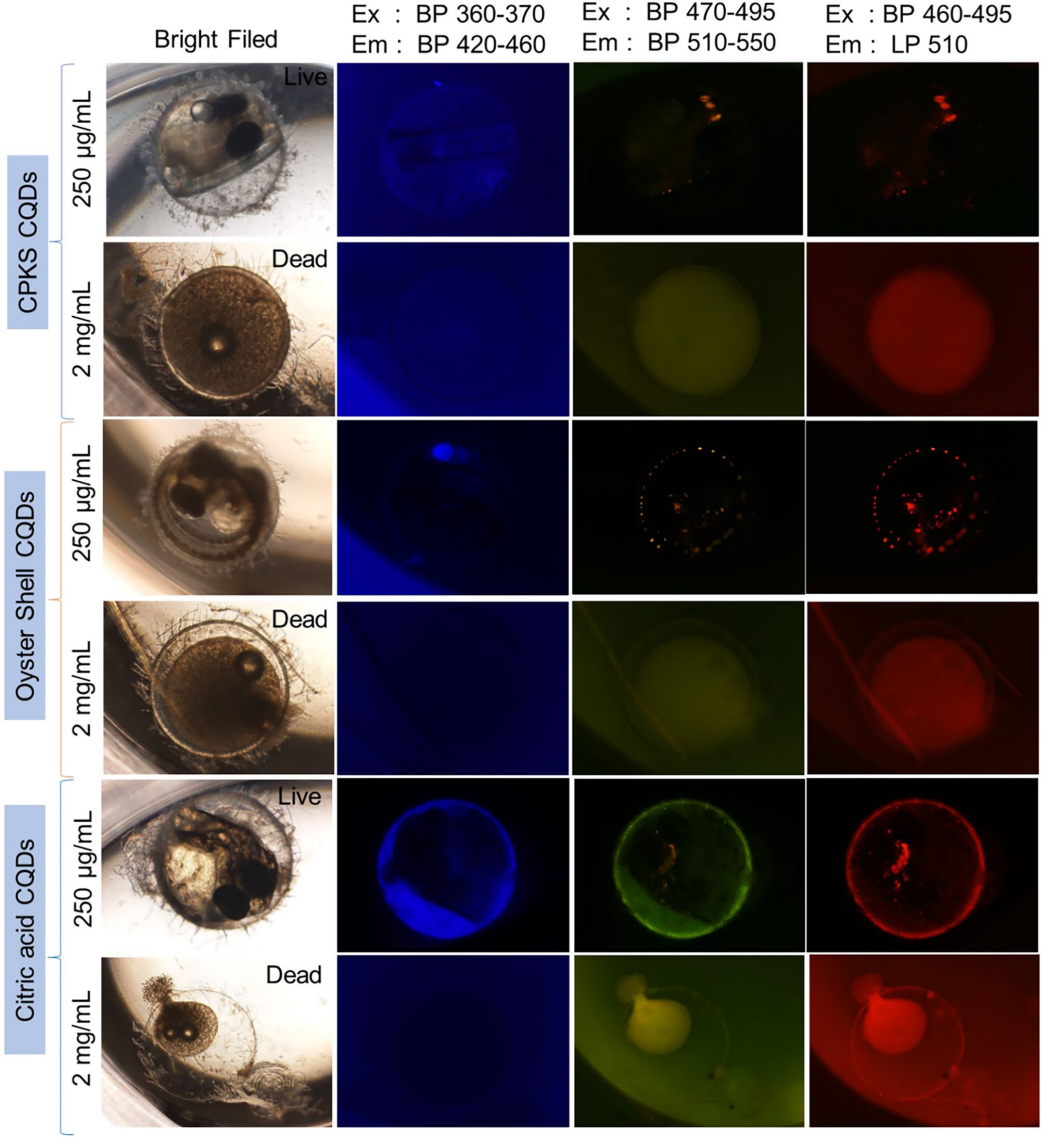
Safety of CPKS, oyster shell, and citric acid based CQDs with Japanese medaka fish eggs: 250 µg/mL is safe, while 2 mg/mL is lethal.

For the in vivo experiments, we investigated CQDs uptake in the fish *Oryzias latipes* model during its early developmental stages. At first, the fertilized eggs were taken from the breeding tank and then seeded onto the 96-well plates with 200uL of DEPC (diethylpyrocarbonate) treated nuclease-free water. Following 24 h incubation, eggs were treated with CQDs at 250 μg/mL and 2 mg/mL concentrations. Confocal fluorescence microscopy was then performed with three different channels to see the effect of CQDs absorption by the eggs. According to Fig. 7, we can spot the fluorescence of three biodots marking their presence in eggs (day 7) without any artificial vectors or chemical transfection agents. By comparing the yellow and red channels to the blue channels, we can accurately determine the color of the CQDs. The results show that even at 250 μg/mL, the fish embryo treated with CQDs displayed no abnormalities. Notably, a QD concentration of 2 mg/mL is detrimental to the survival of fish embryos. Therefore, it is crucial to maintain the concentration below this level to ensure the proper development and survival of the embryos. According to the study by *Kwok *et al*.,* the process of absorption of silver nanoparticles by medaka larvae is predominantly through the skin surface and gills^32^. The research further suggests that this absorption mechanism is due to the small size of the nanoparticles, which allows them to penetrate the skin surface of the larvae easily. The study sheds light on the mode of absorption of silver nanoparticles and highlights the potential risks associated with exposure to such nanoparticles, which can adversely affect the aquatic environment and organisms. *Kashiwada *et al. have conclusively demonstrated that the absorption of nano-sized particles in fish eggs and fry(larvae) is majorly through accumulation or adsorption^33^. Medaka eggs likely take up CQDs through adsorption or gill bioaccumulation. It is crucial to emphasize that CQDs are entirely non-toxic, setting them apart from silver nanoparticles. This feature guarantees the safety of both living organisms and cells.

### Summary

Using biomass-derived waste materials that act as precursors, we synthesized three kinds of CQDs: palm kernel shells, oysters, and citric acid (CA). These innovative methods provide a sustainable solution for waste management and offer a cost-effective and eco-friendly approach to producing high-quality CQDs. Afterward, our team performed fluorescent imaging experiments to determine cellular uptake and efficiency. We carried out rigorous cell cytotoxicity studies, which confirmed the non-toxic behavior of our quantum dots, as there was no cytotoxicity in the first 24 h of incubation. However, after 48 h, we observed a significant level of toxicity, mainly for hiPSc cell culture when treated with citric acid, which resulted in 80% cytotoxicity. Compared with citric acid CQDs, biomass-derived CQDs exhibited minimum toxicity within 24 h (day one). However, for the next 24 h (day two), oyster CQDs displayed a notable level of toxic behavior at higher concentrations when employed on HeLa and hiPSc cultures. This information provides valuable insights that can guide further research toward identifying safer and more effective alternatives to CA-derived CQDs. Furthermore, after performing the cellular fluorescence imaging microscopy studies, we discovered that the CQDs can enter live human dermal fibroblasts, HeLa cells, hiPSc cells, and cardiomyocytes without altering their cellular morphology. Moreover, based on the fluorescence intensity, we could differentiate the untreated (healthy) and inflammed chondrocyte cells with these CQDs. Furthermore, the gene expression profile using qPCR analysis suggests that there is a notable alteration in the inflammatory markers, particularly IL-6, whilst using CA-derived CQDs. Deciphering the mechanism behind how CQDs could simultaneously localize and alter the inflammatory markers could advance their design as novel theranostics for inflammation and immune-related diseases.

### Outlook

Citric acid-based CQDs possess unique properties, making them candidates for reducing inflammation. These CQDs have antioxidant properties that help scavenge reactive oxygen species (ROS), neutralize them, and prevent oxidative damage to cells, thereby reducing inflammation. In addition, CQDs can affect immune cell function and cytokine production. They may decrease the secretion of pro-inflammatory cytokines (such as TNF-α and IL-6) while increasing the production of anti-inflammatory cytokines (such as IL-10), creating a balance of immune responses that affect inflammation. Studies have shown that citric acid-based CQDs can downregulate the endogenous expression of inflammatory marker genes like IL-6 in cell-culture assays and animal models of inflammation^34^. Citric acid-based CQDs may directly inhibit inflammatory pathways by interfering with key signaling pathways, including the NF-κB and MAPK pathways^35^. This alteration of endogenous expression of pro-inflammatory markers may decrease the production of inflammatory mediators.

CQDs synthesized from biomass-waste-based sources offer various advantages as theragnostic agents. They are biocompatible and non-toxic, making them safe for biological studies. This feature is crucial for translational applications as it reduces the harmful effects on cells and organisms. Confocal microscopy studies have shown that these CQDs have the potential for targeted organelle bioimaging and immune system diagnosis studies. Imaging studies can aid in assessing the efficacy and safety of early detection and monitoring of disease progression, leading to better patient outcomes^36^. In conclusion, biomass-derived CQDs can serve as multifunctional theragnostic agents, combining diagnostic imaging capabilities with therapeutic interventions for immune system-associated diseases^37^. Furthermore, our studies showed that CQDs could be safe in Japanese medaka fish eggs, and the results verified their biocompatibility when used at concentrations below 2 mg/mL. Accordingly, they could also be harnessed for potential practical applications to monitor water quality parameters in fish farming conditions and detect pollutants in wastewater.

### Supplementary Information

Supplementary Figures.

## Data Availability

The findings of this study are supported by data that can be made available upon request.
