# Supplementary material for: Biomass-derived carbon dots as fluorescent quantum probes to visualize and modulate inflammation
Source: Sci Rep. 2024 Jun 3;14:12665. doi: 10.1038/s41598-024-62901-7 (PMC11148068; doi:10.1038/s41598-024-62901-7)
Supplement: Supplementary file 1 — Supplementary Figures. [file 41598_2024_62901_MOESM1_ESM.pdf]

Supporting Information

Biomass-Derived Carbon Dots as Fluorescent Quantum Probes to Visualize and Modulate Inflammation

Mahima Kumar[a d], Shanmugavel Chinnathambi[a*], Noremylia Mohd Bakhori[b], Norhidayah Abu[b,c*], Fatemeh Etezadi[a], Vaijayanthi Thangavel[a], Daniel Packwood[a], Easan Sivaniah[a d] and Ganesh N. Pandian[a*]

[a]*Institute for Integrated Cell-Material Sciences, Institute for Advanced Study, Kyoto University, Kyoto, 616-8510 Japan*
[b]*Advanced Materials Research Centre (AMREC), SIRIM Berhad, Lot 34, Jalan Hi-Tech 2/3, Kulim, Hi-Tech Park, 09000, Kulim, Malaysia*
[c]*Department of Medical Microbiology & Parasitology, School of Medical Sciences, Universiti Sains Malaysia, Health Campus, Kubang Kerian 16150, Kelantan, Malaysia*
[d]*Department of Molecular Engineering, Graduate School of Engineering, Kyoto University, Nishikyo-ku, 615-8510 Kyoto, Japan*

Corresponding email address: chinnathambi.shanmugavel.8s@kyoto-u.ac.jp

nhidayah@sirim.my; namasivayam.ganeshpandian.5z@kyoto-u.ac.jp

Functional groups confirmation using Fourier-transform infra-red spectroscopy

The FTIR spectra of the CQDs were displayed in Figure S1 to identify the various surface functional groups present on the CQDs. The excellent water solubility is explained by the FTIR peaks in the 3000–3500 cm^{-1} region, which are related to the hydrophilic groups O-H and N-H of primary aliphatic amines. The peak at 1600 cm^{-1} is due to the existence of the $-NH_2$ scissoring vibration of a primary amine, while the absorption bands at 2850-3000 cm^{-1} represent the C-H stretching vibration (sp^3) attributed to CH_2 groups. The medium peak at 1420 cm^{-1} was attributed to the OH group, while the N-C bonds caused the peak at 1350 cm-1. Moreover, the stretching vibrations of C-OH and the out-of-plane bending modes of C-H groups could be to blame for the peaks in the fingerprint region between 700 and 900 cm^{-1}. Indicating the presence of amine in the EDA and their capacity to dissolve in water, all CQDs had the hydroxyl-functional group (-OH stretching) and amino-functional group (N-H stretching).

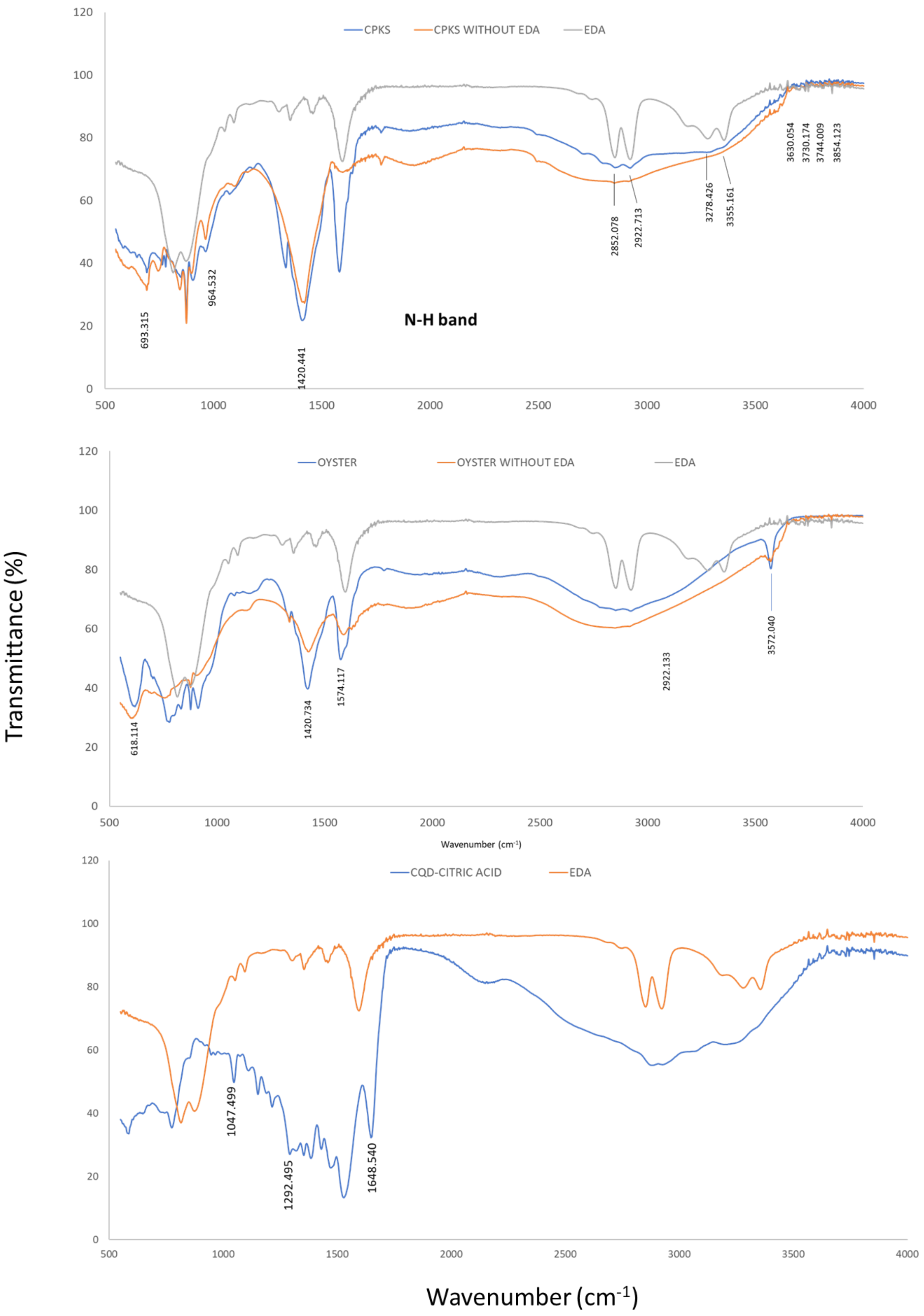

Figure.S1 FTIR characterization of carbon quantum dots from three sources: CPKS (A), oyster shell (B), and citric acid (C).

XPS analysis

Figure S2 shows the binding energy and chemical makeup of the produced CQDs as determined by the surface-sensitive XPS approach. The three distinct peaks in the XPS spectra, which were deconvoluted, showed that oxygen, carbon, and nitrogen made up most of the CQDs. They were attributed to C1s, N1s, and O1s, respectively, for these peaks at 285, 399, and 531 eV. It was reported that the nitrogen that is bound to carbon can enhance the emission of nanoparticles. The deconvoluted peaks of the C1s denoted π-bonded indicate that the carbon atoms from the C-C/C=C, C-N/C-O, and C=O groups are sp2 and sp3 bound. The peaks of O1s are ascribed to the binding energies of C=O and C-O, and the peak of 535 eV indicates the oxygen atom in their moieties like H_2O and OH as the binding energy of water was sensitive to total surface coverage. The peaks of N1s reflect pyrrolic N and amine groups. The pyrrolic N, produced via the dehydrolysis interaction between carboxyl and amine groups, is the primary source of N in the CQDs as they were initially created. According to earlier findings, the pyrrolic N is thought to boost the electronic cloud density of CQD surfaces, thus enhancing luminescence efficiency. These CQDs produced had a multitude of O- and N-related functional groups responsible for their remarkable water solubility and could be strongly correlated with their fluorescence emission.

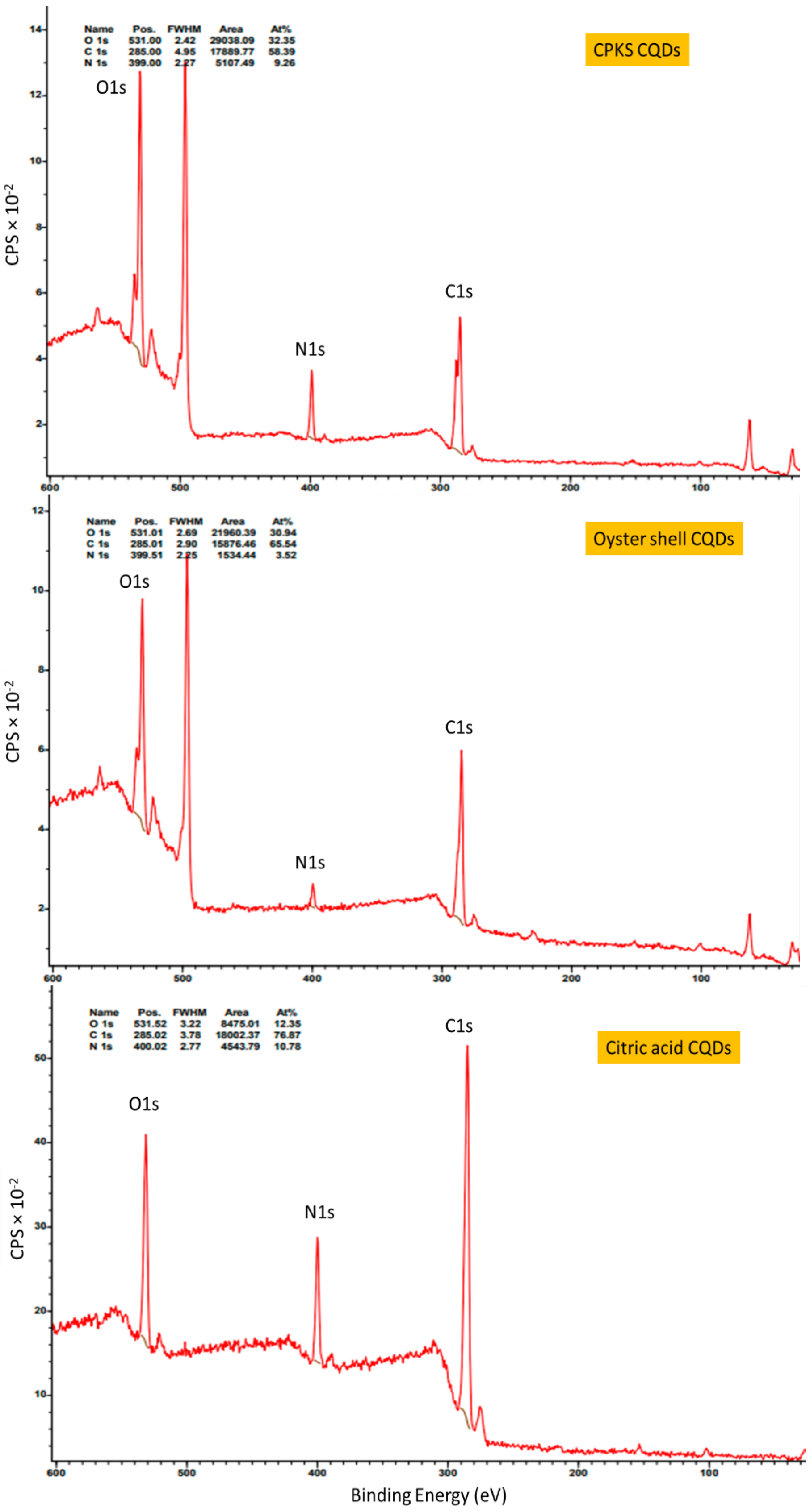

Figure.S2 XPS full-survey spectrum of CQDs from CPKS, Oyster shell and citric acid CQDs.

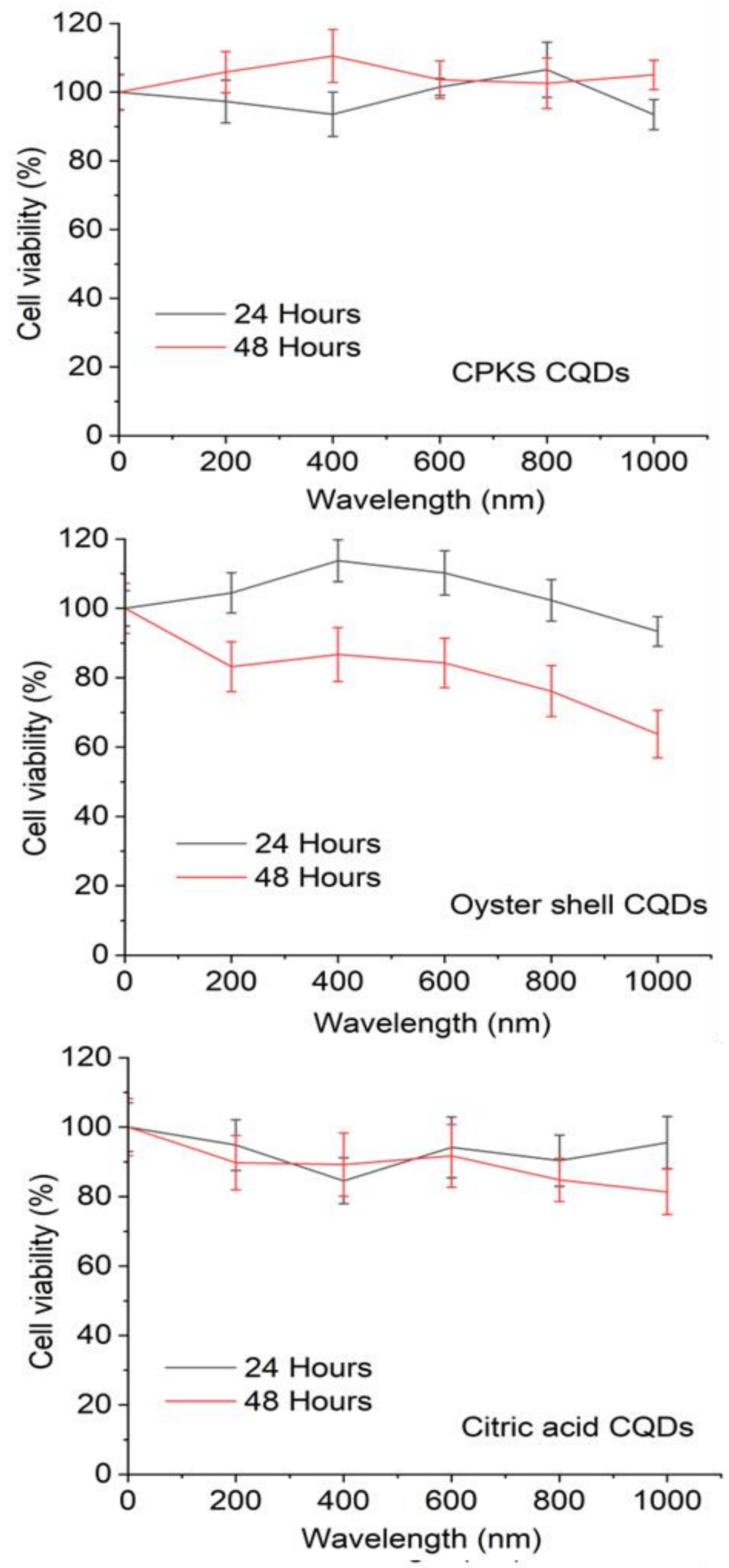

Figure.S3 Cell viability assay using CPKS, oyster shell, and citric acid based CQDs with HeLa cells.

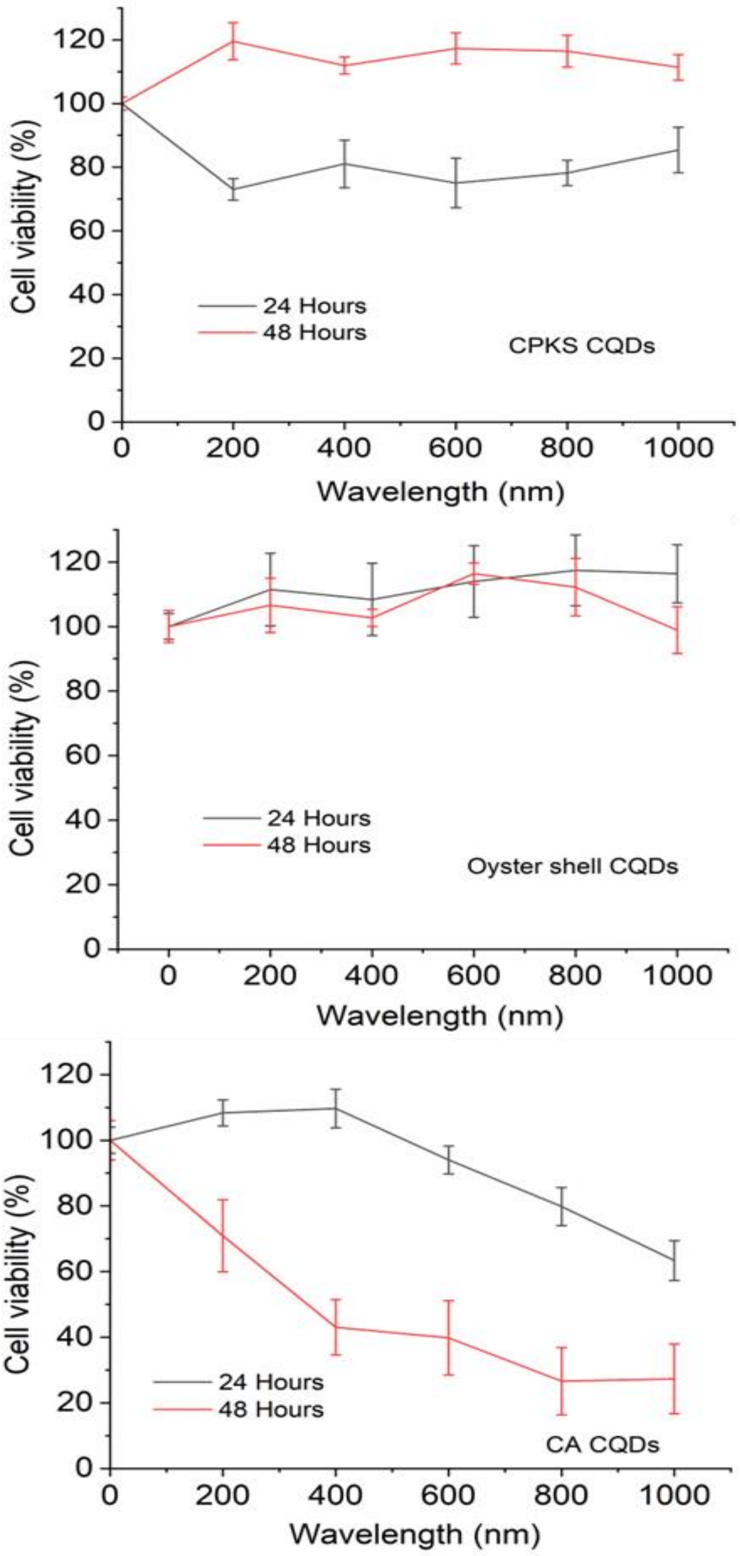

Figure.S4 Cell viability assay using CPKS, oyster shell, and citric acid based CQDs with iPS cells.

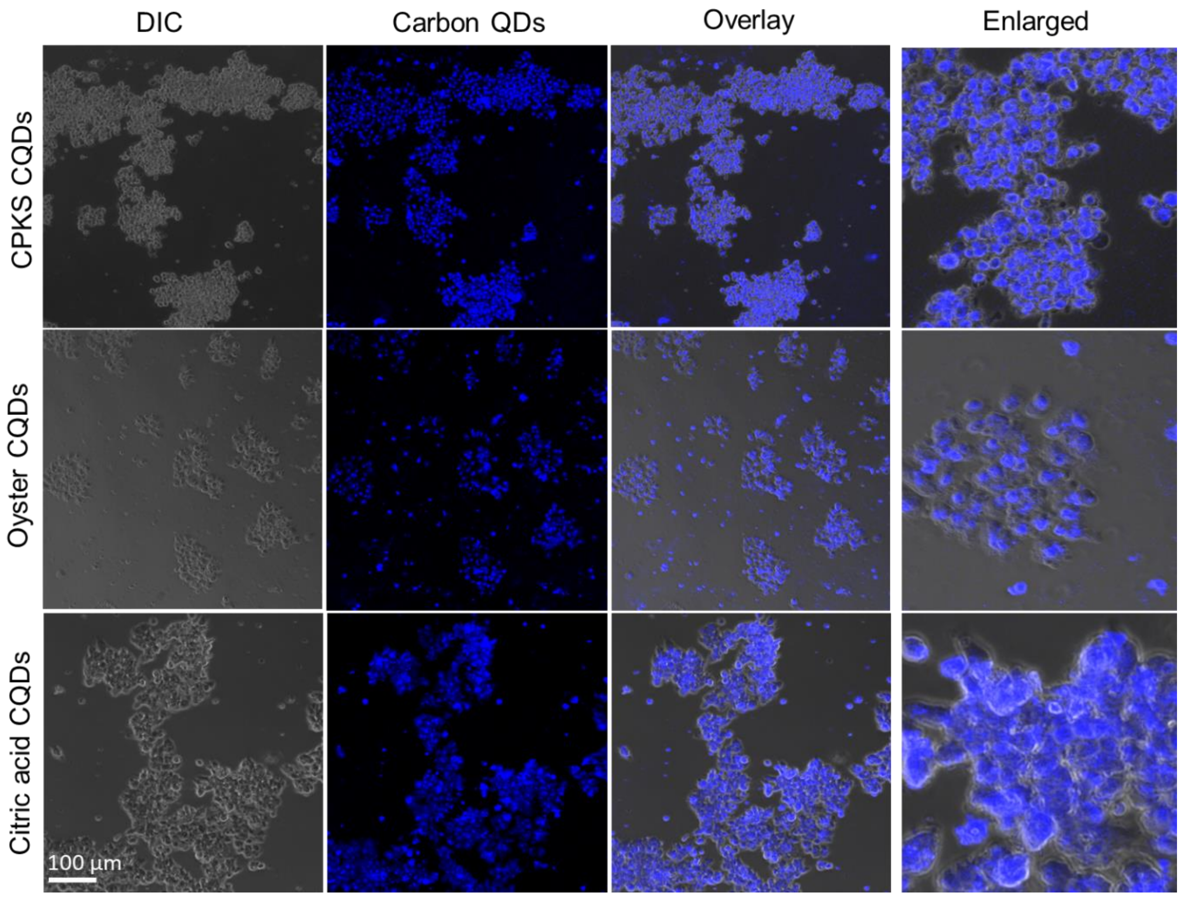

Figure.S5 CPKS, oyster shell, and citric acid based CQDs to perform in vitro imaging experiments with iPS cells.

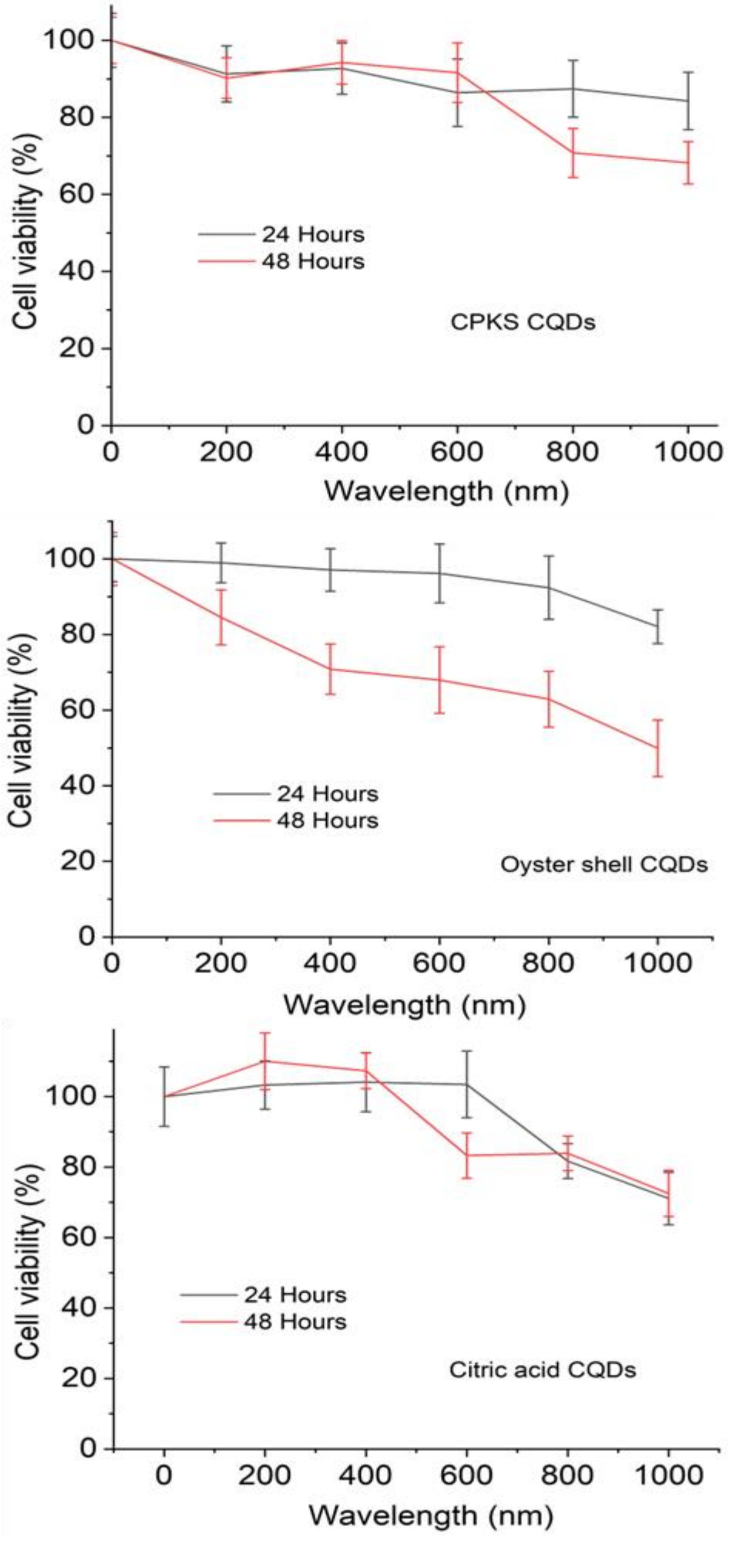

Figure.S6 Cell viability assay using CPKS, oyster shell, and citric acid based CQDs with cardiomyocyte cells.

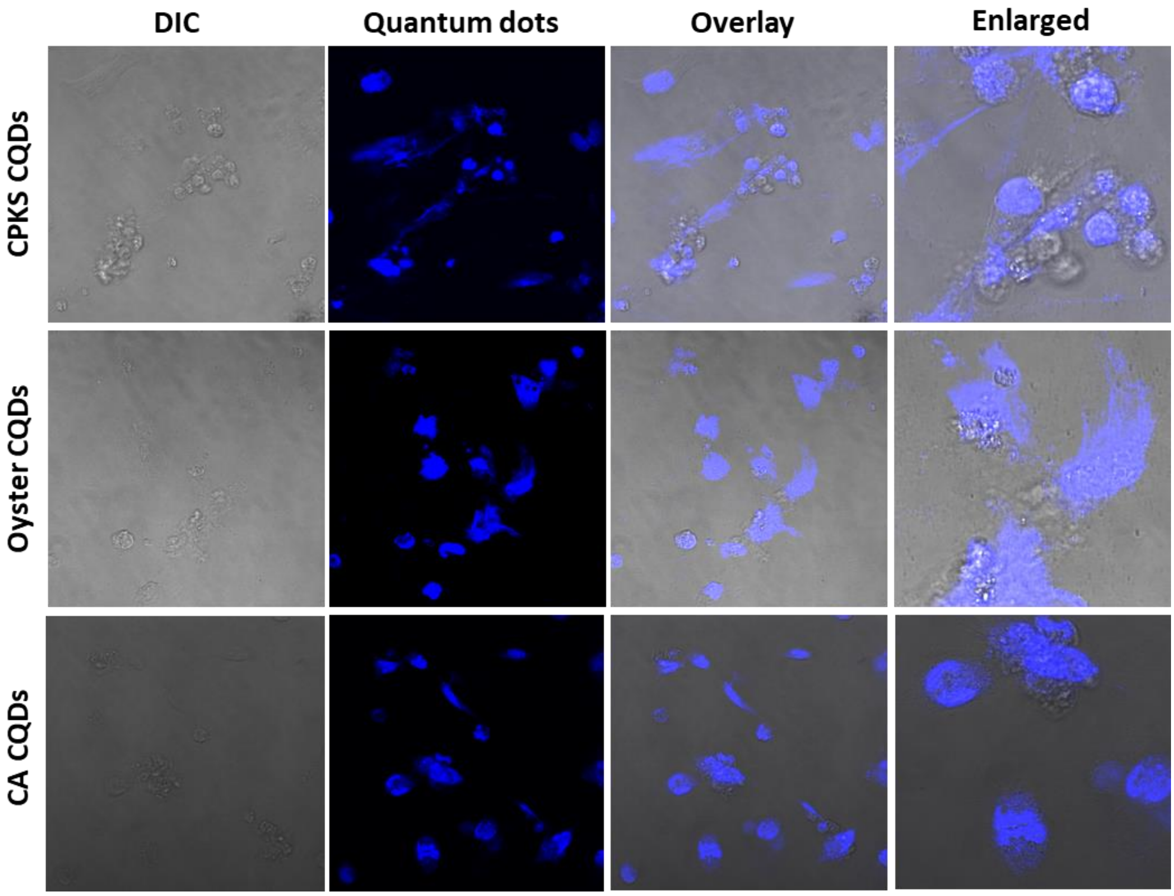

Figure.S7 CPKS, oyster shell, and citric acid based CQDs to perform in vitro imaging experiments with cardiomyocyte cells.
